# Detection and Quantification of Soil-Borne Wheat Mosaic Virus, Soil-Borne Cereal Mosaic Virus and Japanese Soil-Borne Wheat Mosaic Virus by ELISA and One-Step SYBR Green Real-Time Quantitative RT-PCR

**DOI:** 10.3390/v16101579

**Published:** 2024-10-08

**Authors:** Kevin Gauthier, Claudia Janina Strauch, Sabine Bonse, Petra Bauer, Carolin Heidler, Annette Niehl

**Affiliations:** 1 Julius Kühn Institute (JKI)-Federal Research Centre for Cultivated Plants, Institute for Epidemiology and Pathogen Diagnostics, Messeweg 11-12, 38104 Brunswick, Germany; 2 Agroscope, Department of Plant Breeding, Route de Duillier 60, 1260 Nyon, Switzerland

**Keywords:** virus detection method, bipartite virus, quantitative real-time RT-PCR, *Furovirus*, *Polymyxa graminis*, soil-borne wheat mosaic virus, soil-borne cereal mosaic virus, Japanese soil-borne wheat mosaic virus, Tm calculation, ELISA

## Abstract

Furoviruses are bipartite viruses causing mosaic symptoms and stunting in cereals. Infection with these viruses can lead to severe crop losses. The virus species *Furovirus tritici* with soil-borne wheat mosaic virus (SBWMV), *Furovirus cerealis* with soil-borne cereal mosaic virus (SBCMV) and *Furovirus japonicum* with Japanese soil-borne wheat mosaic virus (JSBWMV) and French barley mosaic virus (FBMV) as members are biologically and genetically closely related. Here, we develop SYBR green-based real-time quantitative RT-PCR assays to detect and quantify the RNA1 and RNA2 of the three virus species. Using experimental data in combination with Tm-value prediction and analysis of primer and amplicon sequences, we determine the capacity of our method to discriminate between the different viruses and evaluate its genericity to detect different isolates within the same virus species. We demonstrate that our method is suitable for discriminating between the different virus species and allows for the detection of different virus isolates. However, JSBWMV RNA1 primers may amplify SBWMV samples, bearing a risk for false positive detection with this primer. We also test the efficiency of polyclonal antibodies to detect the different viruses by ELISA and suggest that ELISA may be applied as a first screening to identify the virus. The real-time qRT-PCR assays developed provide the possibility to screen for quantitative disease resistance against SBCMV, SBWMV and JSBWMV. Moreover, with our method, we hope to promote research to unravel yet unresolved questions with respect to furovirus–host interaction concerning host range and resistance as well as regarding the role of multipartite genomes.

## 1. Introduction

Soil-borne wheat mosaic virus (SBWMV) infections were reported for the first time in the USA by McKinney and collaborators [1,2], who observed mosaic symptoms in wheat plants in the year 1919. The rod-shaped structure of SBWMV viral particles [3] and their transmission by the soil-borne plasmodiophorid *Polymyxa graminis* [4] led to the classification of SBWMV into the *Furovirus* (for fungus-borne, rod-shaped virus) genus [5]. Furoviruses are positive-sense single-stranded RNA viruses containing an RNA1 and an RNA2 genome encapsidated into separate particles [6]. RNA1 encodes two replication proteins, where the longer one is produced as a translational read-through of a leaky stop codon [7], an RNA-dependent RNA polymerase and a movement protein. RNA2 encodes a coat protein, a minor coat protein, a coat–protein read-through and a silencing suppressor [6,7,8]. After the identification of SBWMV in the USA, further reports identified the virus in Japan in 1927 [9], in Europe by the end of the 1950s [10] and in China by the end of the 1970s [11]. *Furovirus* classification was extensively discussed in the literature and has drastically changed since its first description. During more than 40 years, all isolates were classified as strains of *soil-borne wheat mosaic virus* and were named according to their geographical locations, i.e., Chinese wheat mosaic virus (CWMV), European wheat mosaic virus (EWMV) and Japanese soil-borne wheat mosaic virus (JSBWMV) [12,13,14]. Although initially clear, the geographical distribution of *soil-borne wheat mosaic virus* strains is today blurrier as SBWMV was reported in Germany and JSBWMV in Germany and France [15,16,17]. In addition, complementation experiments using RNA1- and RNA2-containing particles of EWMV, CWMV and JSBWMV support the conclusion that the viruses are functionally closely related [3]. However, comparisons of the nucleotide sequences between SBWMV and EWMV isolates identified in rye and between SBWMV and CWMV (including JSBWMV) highlighted differences exceeding the species demarcation threshold (currently isolates of the same species share at least 75% nucleotide identity for RNA1 and 80% nucleotide identity for RNA2) [18,19,20]. In addition, monoclonal antibodies distinguished EWMV and CWMV from SBWMV, leading to their reclassification as proper species [21]. While the CWMV strain name was kept as a species name and the classification update to the binominal nomenclature resulted in *Furovirus chinense*, EWMV was temporarily renamed soil-borne rye mosaic virus (SBRMV) because of its identification in rye [15]. ICTV finally merged EWMV and SBRMV nomenclatures to propose the species name *soil-borne cereal mosaic virus* (SBCMV) [22]. The update to the binominal nomenclature resulted in the species name *Furovirus cerealis* (https://ictv.global/report/chapter/virgaviridae/virgaviridae/furovirus, accessed on 7 October 2024). Concerning JSBWMV, RNA2 nucleotide sequence analysis suggested an old reassortment between SBWMV and SBCMV, supporting its classification as a strain [16]. However, RNA1 nucleotide sequence analysis indicated a JSBWMV clustering with *Oat golden stripe virus*, separated from both SBWMV and SBCMV [16]. As for SBCMV and CWMV, this observation led to its recognition as a different species rather than an SBWMV strain [23]. An intermediate species designation as *Soil-borne barley mosaic virus* was proposed for isolates initially identified on barley [16,17,24]. However, nucleotide analysis between isolates from barley in France and Germany and JSBWMV showed sequence identities for RNA1 and RNA2 of above 75% and 80%; therefore, they were identified as strains of *Japanese soil-borne wheat mosaic virus* [12,25] in the new binominal nomenclature *Furovirus japonicum*. Biologically, SBWMV, SBCMV, CWMV and JSBWMV have very similar host ranges, naturally infecting *Poaceae* species. All these viruses are able to infect wheat. Additionally, isolates of *Furovirus cerealis* infect rye and triticale, isolates of *Furovirus chinense* and *Furovirus japonicum* infect barley and isolates of *Furovirus tritici* infect rye, triticale and barley [12,14,26]. Infections by furoviruses typically cause mosaic symptoms, yellowing of the leaves, light stunting and the appearance of yellow patches at the field scale. Infection can lead to important yield losses [14,24,27,28]. As suggested by the multiple updates in the classification, the accurate identification and quantification of each species’ RNA is challenging because of their close genetic relatedness. Thus, the scope of our study was to develop a molecular tool for the specific quantitative analysis of the three viruses. Moreover, we evaluated the efficiency of polyclonal antibodies raised against SBWMV and SBCMV to detect SBCMV, SBWMV and JSBWMV. Several molecular methods for the detection and quantification of furovirus species have been described, but most allow for the detection of a single furovirus species in a multiplex with other cereal viruses [29,30,31,32,33]. Here, we describe a SYBR green-based real-time qRT-PCR method for the quantification of the different RNAs of SBWMV, SBCMV and JSBWMV and evaluate its specificity for each virus. The comparable quantification of different furoviruses allows for the analysis of accumulation patterns in different host species and may help to define levels of resistance in different varieties of a crop plant. Moreover, the possibility of quantifying RNA1 and RNA2 allows for elucidating yet unresolved questions regarding the interaction between virus and host and the possibility of recombination in co-infected plants. The use of coinfection and quantification of RNA1 and RNA2 may moreover shed light on virus factors determining host specificity and host range.

## 2. Materials and Methods

### 2.1. Virus Infection and Plant Material

Seeds of the susceptible wheat cultivar *Reform* were pre-germinated at room temperature in the dark for three days. The seeds were kindly provided by RAGT (Rodez, France). After three days, the wheat plantlets were transferred into infectious soil from Vatan, France (containing SBCMV), or Elxleben, Germany (containing SBWMV), and cultivated at 14 °C. The absence of the other virus (SBWMV in Vatan and SBCMV in Elxleben) was confirmed by RNA sequencing. The plants were watered with 0.5 L of water twice a week. After twelve weeks of cultivation, the plants grown in soil from Elxleben and Vatan were sampled, the roots were washed and plant material was stored at −80 °C. Seeds of the barley cultivars Effi and Kathleen were kindly provided by Saatzucht Josef Breun (Quedlinburg, Germany) and Nordsaat (Langenstein, Germany), respectively. After pre-germination at room temperature for three days in the dark, barley plantlets were transferred to infectious soil from Bornum, Germany (containing JSBWMV) and grown at 12 °C in a climate chamber for two months. After two months of cultivation, the plants were carefully removed from the soil, washed under tap water and placed as donor plants into hydroponic culture in Hoagland solution. The hydroponic culture was maintained in the greenhouse at 17 °C. Approximately one-week-old acceptor seedlings were inserted into the hydroponic culture medium (Hoagland solution) and removed after four to five weeks of cultivation. The roots were washed, and the plant material was stored at −80 °C until RNA extraction.

### 2.2. ELISA

Serological analyses were conducted as described previously [34]. Goat anti-rabbit polyclonal antibodies from the JKI collection were used. The SBWMV-derived antibody JKI-PAS-69, the SBCMV-derived antibody JKI-PAS-92 and their respective alkaline phosphatase conjugates [35,36] were used for DAS ELISA. Plant leaf and root samples were ground in the presence of 1:2 *w*/*v* grinding buffer (PBS (137 mM NaCl, 8 mM Na_2_HPO_4_ 12H_2_O, 2.7 mM KCl, 1.5 mM KH_2_PO_4_, pH = 7.4), 0.05% (*v*/*v*) Tween 20, 2% (*w*/*v*) PVP-K15, 31 mM NaN_3_) using grinding bags (Bioreba, Reinach, Switzerland) and a bench drill press (Einhell, Landau an der Isar, Germany). Leaf or root extracts were tested in the presence of polyclonal capture antibodies JKI-AS92 and JKI-AS69 [35,36]. Concisely, the antibodies were diluted 1/100 (*v*/*v*) in a carbonate coating buffer solution (15 mM Na_2_CO_3_, 35 mM NaHCO_3_, 31 mM NaN_3_, pH = 9.6). Then, 100 µL of the dilution was placed into the wells of a Nunc MaxiSorp™ microtiter plate (Thermo Fisher Scientific, Waltham, MA, USA) before an incubation step of 4 h at 37 °C. The plates were washed once with distilled water and twice with PBS–Tween, prepared as described above. Then, 100 µL of the ground samples was pipetted into the pre-coated wells to be incubated overnight at 4 °C. After washing, alkaline phosphatase-conjugated antibodies (JKI-AS92 and JKI-AS69, respectively) were diluted (1/500 (*v*/*v*) for SBWMV, 1/2000 (*v*/*v*) for SBCMV) in conjugate buffer (grinding buffer supplemented with albumin (0.2% *w*/*v*) as a blocking agent). In total, 100 µL of the dilution was added to the test wells prior to incubation at 37 °C for 3 h. Then, the wells were washed as before. After washing, the wells were filled with 100 µL of the substrate solution (p-nitrophenyl-phosphate (1 mg mL^−1^)) diluted in substrate buffer (1 M diethanolamine, 31 mM NaN_3_, pH = 9.8). The plates were incubated for 2 h at room temperature. Finally, the absorbance at 405 nm was measured using a microplate reader (Tecan, Männedorf, Switzerland). The samples were described as positive when their measured OD exceeded two times the negative control with a minimum threshold of 0.1 after blank reduction. The rest of the positive samples were kept at −80 °C for further use. Twenty plants grown in soil from Elxleben, Vatan and the hydroponic culture were tested.

### 2.3. RNA Extraction

Approximately 50 mg of residual plant material from individual ELISA-positive plants was ground using liquid nitrogen and a Tissue Lyser at 30 Hz for 45 s (QIAGEN, Hilden, Germany). RNA from these ELISA-positive samples was used for qRT-PCR. RNA was extracted from the ground samples using NucleoZol (Macherey-Nagel, Düren, Germany) according to the manufacturer’s instructions. Briefly, the material was suspended in the NucleoZol RNA extraction buffer complemented with RNAse-free water (400 µL mL^−1^ extraction buffer). A centrifugation step (20 min, 12,000× *g*) was conducted before precipitation of the RNA with isopropanol. After ten min of incubation at room temperature, another centrifugation step was conducted. RNA was washed with 500 µL 70% ethanol, followed by centrifugation (3 min, 8000× *g*). After removing the residual ethanol by pipetting, pellets were dried at room temperature for 10 min. The RNA pellet was dissolved in 30 µL of RNAse-free water. The quantity and quality of the extracted RNA were assessed by measuring the OD at 260 and 280 nm (Nanodrop^TM^, Thermo Fisher Scientific).

### 2.4. Primer Design

RNA1 and RNA2 sequences for SBWMV, JSBWMV and SBCMV were retrieved from public databases (National Center for Biotechnology Information, Table S1). Sequences were compared by MUSCLE (Multiple sequence comparison by log-expectation) alignment using the tool provided by the European Bioinformatics Institute (available at https://www.ebi.ac.uk/, accessed on 7 October 2024. For all viruses, cloning and qPCR primers were designed in zones conserved within species genomes and amplified a region that exceeded the beginning of the end of an open reading frame (ORF) (Tables S2 and S3). Primer pair properties (melting temperature, proportion of triple hydrogen bond-binding bases, influence of GC clamps on the melting temperature, risk of secondary structure in the primer, self- and cross-complementarity) were estimated using the online tools provided by Thermo Fisher Scientific (Multiple Primer Analyzer, available at https://www.thermofisher.com/, accessed on 7 October 2024) and Sigma-Aldrich (Saint-Louis, USA, available at http://www.oligoevaluator.com/, assessed on 7 October 2024). Alignments were displayed using the alignment viewer Jalview.

### 2.5. Cloning of the Virus Sequences for Standard Curve Generation

Reverse transcription was performed on fifty-fold diluted RNA. In total, 1 µL of the diluted RNA was used as a template for cDNA-synthesis by using the ProtoScript^®^II Reverse Transcriptase Kit (New England Biolabs, Ipswich, MA, USA), according to the manufacturer’s instructions. First, 1× ProtoScript^®^II reaction buffer, 10 μL of random primers (final concentration of 2 µM), 2 μL DTT, 20 U ProtoScript^®^II Reverse Transcriptase (New England Biolabs) and 125 μmol of each dNTP (12.5 µM) were combined in a 20 µL reaction mixture. Then, reverse transcription was performed in a FlexCycler PCR System (Analytik Jena, Germany) with the following protocol: 25 °C for 5 min, reverse transcription at 42 °C for 1 h followed by a step of 20 min at 65 °C for inactivation of the enzyme. The obtained cDNA was then used as the template (2 μL) in a 20 μL polymerase chain reaction (PCR) mix containing 500 nmol of the respective primer set to detect SBWMV, SBCMV or JSBWMV RNA1 or RNA2 (Table S2), 125 μmol of each dNTP, 4 μL of 5× buffer and 0,5 U One Taq polymerase (New England Biolabs). The samples were placed in a FlexCycler with the following cycle parameters: 5 min at 94 °C followed by 35 cycles of 30 s at 94 °C, 1 min at 50 °C and 2 min at 68 °C concluded with 10 min at 68 °C for final extension. The amplification products were gel-purified using the NucleoSpin^®^ Gel and PCR Clean-up kit (Macherey-Nagel) according to the manufacturer’s instructions. The purified PCR products were ligated into the pDrive vector (QIAGEN, Venlo, The Netherlands) or, for SBCMV RNA2, the pGEM-T (Promega, Madison, WI, USA) by TA cloning. Plasmids were then transformed into *Escherichia coli* DH5α and grown in the presence of the appropriate antibiotic resistance. The presence of inserts was confirmed by colony PCR using M13 primers. Colonies possessing the plasmid with the insert were transferred into liquid LB-medium and cultivated at 37 °C overnight. Plasmids were extracted using a Nucleo Spin^®^ Plasmid kit (Macherey-Nagel), and the insert was confirmed by Sanger sequencing (Eurofins MWG, Ebersberg, Germany). Standard curves were prepared from plasmid DNA by serial dilutions.

### 2.6. One-Step Real-Time qRT-PCR

The identity and quantity of the furoviruses were assessed using one-step real-time reverse transcription (RT)-PCR. One-step real-time RT-PCR was performed with fifty-fold diluted total RNA of the root or leaf samples. First, 1 μL (around 10 ng RNA) of the diluted RNA was added to the reaction mix containing 500 nmol of each primer (Table S3), 20 U ProtoScript^®^II Reverse Transcriptase and Luna^®^ Universal qPCR Master Mix (New England Biolabs) in a 10 μL reaction volume. Then, real-time RT-PCR was performed using a qTower 2.2 (Analytik Jena) with the following protocol: reverse transcription at 42 °C for 10 min, 95 °C for 10 min followed by 40 cycles of 15 s at 95 °C and 15 s at 58 °C before measurement of the melting curves (6 s ramp between 60 °C and 95 °C with an increase of 1 °C at each step). An automatic threshold setting was applied to determine Ct values. The shape of every melting curve was examined using the provided evaluation software (qPCRSoft, Analytik Jena). The identity of the amplicon was assessed using high-resolution melting (HRM) analysis and primer alignment with the target sequence (see paragraph below). Coefficients were calculated from cumulated standard curves (five per standard curve). The efficiencies of the PCR were calculated using the following formula: PCR efficiency = (10^−1/slope^ − 1) × 100 [37]. Each sample was analyzed in two technical replicates.

### 2.7. In-Silico High-Resolution Melting (HRM) Analysis and Estimation of Primer Genericity and Specificity

SBWMV, SBCMV and JSBWMV sequences were retrieved from public databases. Melting temperatures from the target amplicons were calculated using the online tool uMELT [38]. The model of Blake and Delcourt [39] was selected to predict amplicon thermal stability with a concentration of free magnesium of 2 mM and a concentration of monovalent cation of 5 mM. The assessment of primer specificity for each virus and primer genericity (i.e., the ability to identify and amplify diverse virus isolates within the same virus species) was performed by calculating the melting temperature for two (specificity prediction) or three (genericity prediction) different isolates for each virus species. The analysis was completed by counting the number of mismatches located in the five 3′ proximal nucleotides of the primers after alignment with their target sequence. The number, location and identity of the mismatches have an important impact on both melting temperature and threshold cycle (e.g., [40,41,42,43,44]). For these reasons, we suggested a 1 °C difference in Tm between the primer-specific target determined with the standard curve and the calculated Tm as the species demarcation level in our HRM study. Samples producing an amplicon with a Tm deviating more than 1 °C from the experimentally determined Tm using the standard curve were considered negative in HRM. Vice versa, samples were considered positive in HRM when the Tm deviated less than 1 °C from the experimentally determined Tm. For the experimental data, the Ct values were taken as an additional criterion to determine if a PCR was positive or negative. A PCR result was considered negative if ΔTm (Tm_sample_ vs. Tm_standard_) > 1 °C and/or Ct > 35 or at least 5 Ct values higher than the last standard curve data point.

### 2.8. Statistical Analysis

Principal component analysis (PCA) was performed on the normalized optical densities obtained by ELISA to cluster the different tested samples (infected by SBWMV, SBCMV and JSBWMV). Linear regression was performed to link the target concentration and the number of amplification cycles in qPCR. In addition, 95% confidence intervals were calculated around the calculated average estimated value. All statistical analyses were performed using R software version 3.6.3 [45]. Figures were drawn using the ggplot2 v3.4.3 package [46].

## 3. Results

### 3.1. Differential Reaction of SBCMV, SBWMV and JSBWMV to Polyclonal Antibodies

**Figure 1:**
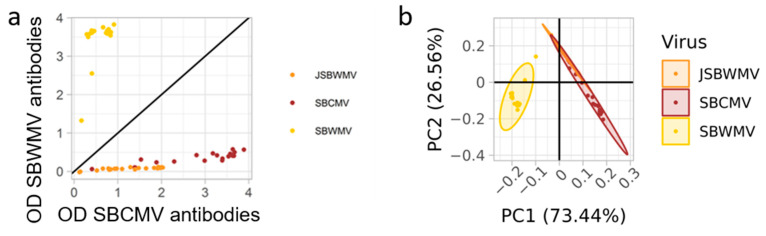
Results of ELISA using polyclonal antisera derived from SBCMV and SBWMV virions. (**a**) Optical densities (ODs) at 405 nm measured for SBWMV, SBCMV and JSBWMV after blank removal. The black line represents the equation y = x. (**b**) Principal component analysis (PCA) performed on ELISA optical densities at 405 nm obtained with SBWMV and SBCMV antibody sets. Confidence intervals are represented by ellipses.

Wheat plantlets grown in SBWMV- and SBCMV-containing soil, respectively, were analyzed for their SBWMV and SBCMV content, while the roots of barley plantlets grown in hydroponic culture containing JSBWMV were analyzed for their JSBWMV content. Antibodies raised against SBWMV identified 17 infected out of the 20 plants grown in the soil from Elxleben (containing SBWMV) with an average optical density of 3.43 ± 0.61. (Figure 1a). Similarly, antibodies raised against SBCMV identified 19 infected out of 19 plants grown in the soil from Vatan containing SBCMV (average OD: 2.81 ± 1.02) and 20 out of 20 in plants from the hydroponic culture containing JSBWMV (average OD: 1.22 ± 0.62). Antibodies raised against SBWMV identified 89.5% (17/19) of the plants infected with SBCMV as positive but with a significantly lower OD compared with the OD, with which the SBWMV-derived antibodies detected SBWMV (p = 2 × 10^−16^, average OD: 0.38 ± 0.12). Only 15% (3/20) of the JSBWMV-infected plants were identified using the antibodies raised against SBWMV, with significantly lower OD compared with those for which the SBWMV-derived antibodies detected SBWMV and SBCMV (p = 2 × 10^−16^ and 0.015, respectively, average OD: 0.110 ± 0.003). Vice versa, all SBWMV-infected plants were detected using the antibodies raised against SBCMV, but the optical densities were significantly lower compared with those for which the SBCMV-derived antibodies detected SBCMV (p = 4.85 × 10^−13^, average OD: 0.56 ± 0.24). Using the antibodies raised against SBCMV, the samples infected with JSBWMV exhibited higher OD than the samples infected with SBWMV but lower OD than the samples infected with SBCMV (p = 0.007 and 4.41 × 10^−9^, respectively). As the differences in OD between the viral species for the same antibodies could be explained by a differential accumulation of the viruses, we compared the OD produced for each virus with the two sets of antibodies. SBWMV-infected samples produced higher OD values when tested with the antibodies raised against SBWMV (p = 2 × 10^−16^), while the SBCMV- and JSBWMV-infected samples produced higher OD values when tested with the antibodies raised against SBCMV (p = 1.83 × 10^−12^ and 4.66 × 10^−10^, respectively). We finally conducted a principal component analysis (PCA) after normalizing the OD of the positive samples. Component 1 (PC1), explaining 73.44% of the variance in the OD values, allowed for a clear separation of the SBWMV-infected samples from the SBCMV- and JSBWMV-infected samples (Figure 1b). Component 2, explaining 26.56% of the variance in the OD values, tended to separate JSBCMV from SBCMV but not significantly.

### 3.2. Standard Curves for Real-Time RT-PCR

**Figure 2:**
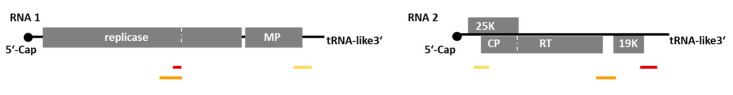
Furovirus genome structure and location of the regions to be amplified by qRT-PCR (not drawn to scale). Different open reading frames (ORFs) with the names of encoded proteins are shown as grey boxes. Dashed lines within the replicase and the CP-RT ORF indicate translational readthrough codons. The relative location of the regions to be amplified with the different primer pairs to detect RNA1 and RNA2 of the different viruses is shown by lines underneath the scheme. SBWMV, yellow; JSBWMV, orange; SBCMV, red.

Amplicons from conserved regions within species were generated with a size between 60 and 150 nucleotides by real-time RT-PCR (Table S3). The amplified regions were designed to cover a region directly translated from genomic RNA or exceeding the end of ORFs to reduce the likeliness of amplifying possible subgenomic RNAs (Figure 2). Concerning RNA1, the region amplified a part of the SBWMV 37 kDa movement protein-coding sequence and the non-coding 3′ extremity of the virus. The region amplified for SBCMV and JSBWMV covered parts of the replicase ORF. Concerning RNA2, the region amplified for SBWMV included a part of the coat protein read-through sequence and genomic RNA, the region amplified for SBCMV covered a part of the silencing suppressor ORF and genomic RNA and the region amplified for JSBWMV covered part of the coding region for the coat protein read-through and the silencing suppressor. Serial dilutions were performed with the plasmids containing target sequences of each RNA of the different virus species with efficiencies between 103 and 107% and R^2^ ≥ 0.977 (Figure S1, Table 1), allowing for the precise quantification of each RNA for the tested species. Using the standard curves, quantification of RNA1 was possible for all virus species between 0.01 and 100 ng of the target µL^−1^. The quantification of JSBWMV RNA2 was conducted in the same concentration range. SBWMV RNA2 was quantified between 0.67 and 100 ng target µL^−1^ RNA, while SBCMV RNA2 was quantified between 0.001 and 5 ng target µL^−1^ RNA (Table 1). It should be noted, however, that the concentration range of the standard curve for detecting the viruses may vary if different matrices are used.

### 3.3. Stability of the Standard Curves in One-Step Real-Time qRT-PCR Using SYBR-Green ^®^

**Figure 3:**
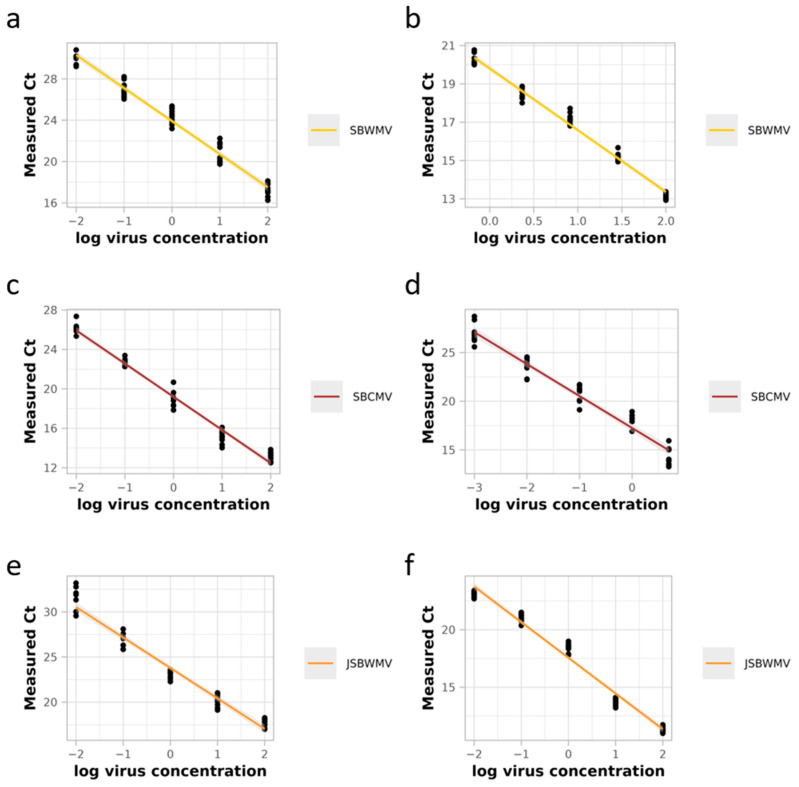
Stability of the standard curves. Five real-time qRT-PCRs were conducted on independent plates with independent dilution series of the plasmids with two technical replicates each to determine the stability of the standard curves for SBWMV RNA1 (**a**) and RNA2 (**b**), SBCMV RNA1 (**c**) and RNA2 (**d**) and JSBWMV RNA1 (**e**) and RNA2 (**f**). Measured Ct: number of amplification cycles required to exceed the detection threshold. Log virus concentration is in ng µL^1^ total RNA. Confidence intervals are based on the Student’s distribution approximation and are represented by grey zones.

The robustness of the assays was assessed in five repetitions of the standard curves on independent plates using independent dilution series of the plasmids (Figure 3). The analysis of the standard curves displayed a slight drop in R^2^ compared with the single-curve R^2^, but it remained high for all standard curves (R^2^ > 0.951, Table 1). In addition, the confidence intervals estimated for the efficiency of the real-time qRT-PCR remained within the recommendation interval (90–110%) of the minimum information for the publication of quantitative real-time PCR experiments (MIQE) Guidelines [47] for both RNAs of all virus species (Table 1).

### 3.4. Test for Specificity

**Figure 4:**
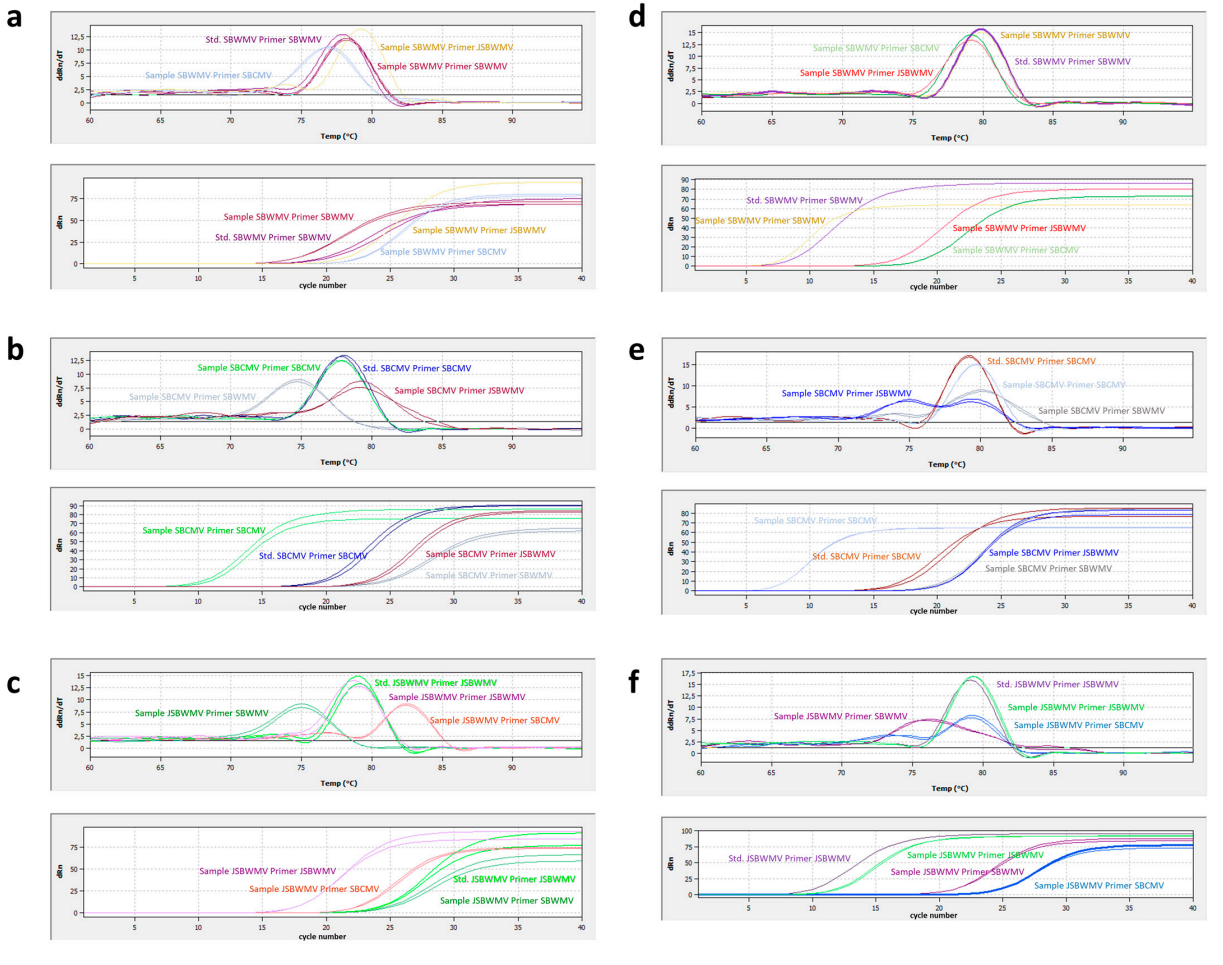
Analysis of qRT-PCR specificity. The specificity of the PCR was analyzed by comparing the melting curves of the PCR amplicons generated by qRT-PCR with the different primers for plant samples with the melting curves generated for the standard DNA (upper panels) and by comparing the Ct values generated by qRT-PCR with the different primers for the plant samples (lower panels). Standards (Stds) were taken from within the range of the standard curves. (**a**) SBWMV amplification, RNA1 primers; (**b**) SBCMV amplification, RNA1 primers; (**c**) JSBWMV amplification, RNA1 primers; (**d**) SBWMV amplification, RNA2 primers; (**e**) SBCMV amplification, RNA2 primers; (**f**) JSBWMV amplification, RNA2 primers. Images of representative samples analyzed by the qPCR soft software version 3.4 (Analytic Jena) are shown. ddRn/dT, derivation of change in fluorescence values over time; dRn, change in fluorescence values.

The PCR test was assayed for specificity by testing whether the primer pairs designed to amplify regions on SBWMV, SBCMV and JSBWMV RNA1 and RNA2 of the different viruses were able to amplify regions within the other two related viruses (Table 2, Figure 4). Whether a PCR was considered positive was based on the criterion that the difference between the Tm obtained with the amplified PCR product from the infected plant material and the Tm obtained with the standard curve was less than 1 °C and the criterion that the Ct value obtained was above 35 or exceeded the Ct value obtained for the most diluted standard curve data point by at least 5 Ct. The PCR test was found to be specific for the detection of SBWMV with SBWMV primers and for SBCMV with SBCMV primers. In addition, JSBWMV was reliably detected with JSBWMV primers; however, a PCR amplicon with a low Ct value was also obtained when an SBWMV RNA1 amplicon was amplified with 1-jSBW-3964-FW and 1SBC-4053-RV primers (Table 2, Figure 4). Because of the ΔTm value, which was only slightly higher than 1 °C, and the low Ct value, this amplicon could be easily misinterpreted as positive. RNA2-specific JSBWMV primers were specific for the detection of JSBWMV.

To further assess the specificity of the PCR, we performed HRM with the following three-step evaluation: (i) establish whether the experimentally determined Tm corresponded to the Tm predicted by HRM, (ii) determine whether the Tm predicted for a PCR using the other related virus as target (e.g., SBWMV primers to amplify SBCMV) could be mistaken for the amplification of the intended target, and (iii) determine via HRM whether the generation of an amplicon during a PCR with a given primer–target combination is realistic based on primer–target mismatch. To determine the effect on the target concentration, we evaluated the impact of the different mismatches on the primers [44]. Depending on the nature and location of the mismatch within the primer sequence, a single mismatch may lead to the absence of amplification. Therefore, the evaluation of the impact of primer–target mismatch constituted the last step of our HRM method. We applied HRM to evaluate the within-species genericity of the PCR and the specificity of the PCR.

### 3.5. Within-Species Genericity of the PCR

The predicted Tm for detecting different isolates of each virus with the corresponding primer pairs deviated only a little from the experimentally determined Tm for detecting the respective virus (Table 2 and Table 3). The difference in Tm was always below 1 °C, except for JSBWMV NC038850, which was predicted not to be detected with JSBWMV RNA1 primers (1-jSBW-3964-FW and 1SBC-4053-RV). The JSBWMV JT-isolate (NC038850) showed some degree of sequence diversity with the experimentally tested Bornum isolate (MN 123252, Figure S2). The amplicon sequence differed in 15 nucleotides between the two isolates, not counting mismatches in the primers. However, as the primer sequence contains no mismatches with the JSBWMV JT-isolate, the isolate is presumably detected and quantified with the primers.

Besides the differences in Tm, mismatches in the five 3′-proximal nucleotides in the primer sequences and the target sequences were also examined. Between the different isolates analyzed for the RNA1 of each virus, only one such mismatch was found in RNA1 (Table 3, Figure S2). This mismatch discriminates between the Nebraska and New York strains of SBWMV investigated here and is located at the 3′ end of the forward primer (1-BWF-6541T, Figure S2). This mismatch (replacement of an A by a G on the fourth to last nucleotide) was determined to have only a minor effect on target amplification [44]. For RNA2, two mismatches within the five 3′ proximal nucleotides were present in the SBWMV primers (2BWF-278) compared with the SBWMV-NY isolate KT736089 sequence (Table 3, Figure S3). These mismatches were predicted to allow for the binding and the quantification of the target, however, at the cost of an increase in the Ct value of around two cycles [44].

### 3.6. Assessment of PCR Specificity by HRM Analysis

HRM analysis was applied to calculate Tm for the amplification products derived from the other related species with a given primer pair designed for one species. The PCR result was estimated based on the rule that the PCR is considered negative when the calculated Tm exceeds the Tm determined experimentally for the primers with their specific target by 1 °C. In addition, the number and effect of mismatches in the five 3′ proximal nucleotides within the primer sequences compared to the virus sequence was estimated.

The predictions with different isolates of the viruses revealed the PCR to be specific for the detection of SBWMV with the SBWMV primers (Table 3), which is consistent with the results obtained experimentally (Table 2, Figure 4). For the detection with primers targeting SBCMV RNA1 (1-SBC-3993-FW and 1-SBC-4053-RV), HRM predicted a false positive PCR result for JSBWMV RNA1 targets based on Tm (Table 3). However, as the forward primers displayed three and four mismatches for NC_038850 and MN_123252, respectively, in the 3′ proximal nucleotides (Figure S4), the PCR with SBCMV primers will likely not amplify JSBWMV RNA1. For the primers targeting JSBWMV RNA1 (1-jSBW-3964_FW and 1-SBC4053-RV), a false positive result was predicted for the amplification of SBWMV-NY (KT736088). This is consistent with the experimentally observed detection of SBWMV with these primers with a low Ct value and a Tm only slightly above the threshold (Table 2, Figure 4). For the amplification of SBWMV RNA2 with primers targeting JSBWMV RNA2 (qSBBMV-ANF and qSBBMV-ANR), a false positive result was predicted (Table 3). However, because of the three mismatches within the five 3′ proximal nucleotides in the qSBBMV-ANR sequence (Figure S5), the primer is unlikely to bind or produce a PCR product. All other predictions yielded the specific result for the detection of the target virus with the corresponding primer.

## 4. Discussion

While ELISA is a cheap and convenient detection method for large numbers of samples, its sensitivity can be limited, and its specificity depends on the virus to be detected, the diversity within the virus species, the relatedness of the virus with other virus species and the quality of the antibodies [48]. In contrast, molecular methods, such as (RT)-PCR, LAMP and real-time (RT)-PCR allow for the sensitive detection of viruses and, in the case of high-throughput sequencing (HTS), the sensitive detection of unknown viruses in a given sample [48]. In the case of HTS and real-time (RT)-PCR, virus amounts in the samples can be quantified, which is specifically important for studies aiming at identifying quantitative resistance genes or mechanisms of infection.

As SBWMV, SBCMV and JSBWMV share the same vector, host species, symptoms and geographical distribution, their biological characterization remains difficult [24,27,31,49]. Here, we developed an SYBR-green-based method for the quantitative analysis of SBCMV, SBWMV and JSBWMV RNA1 and RNA2 and tested its specificity and selectivity using plant samples and prediction methods. Several molecular and serological methods have been developed to detect and quantify *P. graminis*-transmitted viruses. Immunocapture and loop-mediated isothermal amplification (LAMP) methods were developed for the detection and large-scale qualitative analysis of *P. graminis*-transmitted cereal viruses [32,50,51,52]. Moreover, methods for the quantification of SBCMV have been published with the aim to achieve high sensitivity for detection and to develop tools for the quantitative analysis of resistance in wheat and triticale varieties [29,31] and the prevalence of SBCMV in grasses [30]. However, despite this number of methods to detect *P. graminis*-transmitted viruses and to quantify SBCMV, no method has yet been published to quantify both RNAs of SBCMV, SBWMV and JSBWMV. The tool we present here may help answer questions related to virus–host interaction, virus accumulation within host species and the interaction between different *P. graminis*-transmitted viruses within a host plant. Moreover, the method may be useful for the quantification of viruses in different host plant varieties during resistance screening. As furovirus resistance genes confer quantitative resistance, the identification of novel resistance QTLs requires quantitative phenotypic data (e.g., [31,53,54]).

The use of SYBR-green real-time PCR to quantify viruses in plant samples along with the identification of the product using dissociation curves is a method that proved its efficiency and stability in the past [55,56,57,58]. Here, we tested plant material of samples naturally infected with SBWMV, JSBWMV and SBCMV isolates and applied stringent conditions based on ΔTm determined experimentally and in silico via HRM and Ct values for their identification. The primers were developed to amplify conserved regions within species; thus, our method was expected to be applicable for different isolates of the viruses while discriminating between the different virus species. Indeed, except for JSBWMV RNA1 primers (1-jSBW-3964-FW and 1-SBC-4053-RV), which could amplify an SBWMV amplicon; all primer pairs were specific for their target virus species. False positive detection of SBWMV was also predicted for this primer by HRM. The other false positive PCR results predicted based on ΔTm values are unlikely to occur because of important mismatches in the primer sequences (Table 3 and Figures S4 and S5), leading to no or poor binding and PCR amplification and strong increases in Ct values [44]. Within species, the primers displayed high sequence identity to the different target isolates analyzed. Mismatches between the five 3′ proximal nucleotides of the primers and target species impacting primer binding [44] were avoided; hence, only the 1-BWF-6541T primer contained one such mismatch when aligned to the SBWMV-NY sequence (Table 3, Figure S2). Consistently, in silico HRM revealed ΔTm < 1 °C for amplicons from different isolates of the viruses (Table 3). The predicted TMs were consistent with the experimentally determined TMs for the representative isolates.

Compared with other real-time PCR methods to detect plant viruses, our method performs in a good sensitivity range by detecting and quantifying approximately 120 K copies of viral RNA per µL total RNA. WDV, for example, has been described to amplify 14 K copies of viral DNA per µL total DNA [55]. Ratti and collaborators [29] were able to detect SBCMV at a concentration of up to 10^−3^ pg/ng RNA. In both cases, our method is less sensitive by a factor of 10, but for WDV, no RT step is required prior to PCR, and Ratti and collaborators [29] describe a TaqMan-based method. LAMP, in contrast, achieves a much higher sensitivity, of approximately 10 copies of viral RNA [51], but it is a qualitative method only. Thus, overall, at the cost of a slight loss in sensitivity compared with TaqMan methods, our SYBR green-based method has the advantage of high cost efficacy and high flexibility in the applicability for different research questions.

In this study, we also evaluated the reaction of polyclonal antibodies raised against SBCMV and SBWMV for their efficiency in detecting SBWMV, SBCMV and JSBWMV. Serology enabled the separation of SBWMV-infected samples from SBCMV- or JSBWMV-infected samples. However, the JSBWMV-infected samples are serologically not distinguishable from the SBCMV-infected samples with a low virus titer with our polyclonal antibodies. The cross-reaction observed between SBCMV and JSBWMV in ELISA is not surprising as these species share 90.91% identity in their coat protein amino acid sequence (after MUSCLE alignment of the sequences NC_002330.1 and MN123254.1, both available in public databases). As their identity with the SBWMV (NC_002042.1) coat protein amino acid sequence is much lower, reaching only 78.98% (SBCMV) and 81.82% (JSBWMV), it is not surprising that SBWMV antibodies only poorly detect SBCMV and JSBWMV. If high numbers of samples are to be investigated, ELISA tests using polyclonal antibodies could be a first qualitative screening step before differential virus analysis using qRT-PCR.

The data we obtained by evaluating the serological and molecular biological methods to detect the three different virus species *Furovirus tritici*, *Furovirus cerealis* and *Furovirus japonicum* also support the suggestion that JSBWMV is a reassortant of SBWMV and SBCMV [13,19], as JSBWMV cross-reacted with SBCMV in serology (based on the coat proteins expressed from RNA2) and with SBWMV in real-time PCR (for RNA1).

## Figures and Tables

**Table 1:** Assessment of standard curve robustness using SYBR-green one-step qRT-PCR for RNA1 and RNA2 of SBWMV, SBCMV and JSBWMV.

Virus	RNA	Average Efficiency (%)	CI Efficiency (%)	Adjusted R-Square	Concentration Range (ng)
SBWMV	RNA1	105.35	98.5–113.2	0.972	0.01–100
SBCMV	RNA1	98.31	92.6–104.7	0.977	0.01–100
JSBWMV	RNA1	97.7	90.2–107	0.953	0.01–100
SBWMV	RNA2	104.48	99.2–110.3	0.984	0.67–100
SBCMV	RNA2	101.78	92.5–113	0.951	0.001–5
JSBWMV	RNA2	109.97	104.3–116.3	0.975	0.01–100

CI: 95% confidence interval. Five real-time qRT-PCRs with independent dilution series of the targets were performed for each standard curve.

**Table 2:** Assessment of PCR specificity.

Virus Species	RNA	Primer Target Species	Threshold Cycle	Tm	Tm_std_	PCR Result
**SBWMV**	**RNA1**	**SBWMV**	**11.55**	**77.85**	**78**	**Positive**
SBWMV	RNA1	SBCMV	29.26	75.25	78	Negative
SBWMV	RNA1	JSBWMV	14.85	79.3	78	Negative
SBCMV	RNA1	SBWMV	29.29	81.5	77.8	Negative
**SBCMV**	**RNA1**	**SBCMV**	**14.48**	**78**	**77.8**	**Positive**
SBCMV	RNA1	JSBWMV	27.91	80.1	77.8	Negative
JSBWMV	RNA1	SBWMV	30.36	71.5	79.6	Negative
JSBWMV	RNA1	SBCMV	26.49	80.25	79.6	Negative
**JSBWMV**	**RNA1**	**JSBWMV**	**17.07**	**78.85**	**79.6**	**Positive**
**SBWMV**	**RNA2**	**SBWMV**	**7.25**	**79.45**	**79.3**	**Positive**
SBWMV	RNA2	SBCMV	29.32	78	79.3	Negative
SBWMV	RNA2	JSBWMV	24.8	78.3	79.3	Negative
SBCMV	RNA2	SBWMV	18.27	76.6	79.5	Negative
**SBCMV**	**RNA2**	**SBCMV**	**13.5**	**79.9**	**79.5**	**Positive**
SBCMV	RNA2	JSBWMV	22.3	76.4	79.5	Negative
JSBWMV	RNA2	SBWMV	20.07	75.9	78.7	Negative
JSBWMV	RNA2	SBCMV	29.35	78	78.7	Negative
**JSBWMV**	**RNA2**	**JSBWMV**	**10.64**	**78.7**	**78.7**	**Positive**

The specificity of the PCR was assessed with plant samples naturally infected with SBWMV (isolate from Elxleben), SBCMV (isolate from Vatan) and JSBWMV (isolate from Bornum) and primer pairs designed for the detection of each of the viruses. Tm is the melting temperature for the PCR amplicon derived from the amplification of a plant sample using the primers outlined in column 3 of the above table (primer target species); Tm_Std_ is the melting temperature for the PCR amplicon derived from the amplification of the standard DNA with the corresponding primers targeting the virus shown in column 1 (virus species). The PCR result in the rightmost column refers to the virus species to be detected (column virus species). Values for representative samples are shown in the table. The samples were considered negative when the difference between the amplicon melting temperature and the melting temperature obtained for the standard exceeded one degree Celsius. In addition, in samples with a Ct value of more than 35 or with a Ct value exceeding the Ct value produced by the last data point of the standard curves by 5 Ct were considered negative.

**Table 3:** Assessment of PCR specificity based on HRM and sequence alignment.

Accession Number	Virus Species	RNA	Primer Target Species	Calculated Tm	Predicted PCR Result	Mismatch 5 LNT FWD	Mismatch 5 LNT REV	Comments
NC_002041	SBWMV-N	RNA1	SBWMV	77.5	Positive	0	0	
KT736088	SBWMV-NY	RNA1	SBWMV	77.75	Positive	1	0	
NC_002351	SBCMV	RNA1	SBWMV	80	Negative	4	3	
AJ132576	SBCMV	RNA1	SBWMV	80	Negative	4	3	
NC_038850	JSBWMV	RNA1	SBWMV	73.75/77.5	Negative	1	2	Double peak predicted, reverse primer does not match, insertion
MN123252	JSBWMV	RNA1	SBWMV	80.5	Negative	2	2	
NC_002351	SBCMV	RNA1	SBCMV	78	Positive	0	0	
AJ132576	SBCMV	RNA1	SBCMV	78	Positive	0	0	
NC_002041	SBWMV-N	RNA1	SBCMV	76	Negative	2	0	
KT736088	SBWMV-NY	RNA1	SBCMV	76.75	Negative	2	0	
NC_038850	JSBWMV	RNA1	SBCMV	77.75	False positive	3	0	Primer binding and amplicon formation unlikely
MN123252	JSBWMV	RNA1	SBCMV	77.5	False positive	4	0	Primer binding and amplicon formation unlikely
NC_038850	JSBWMV	RNA1	JSBWMV	81.25	Negative	0	0	Specific amplicon formation with deviating Tm
MN123252	JSBWMV	RNA1	JSBWMV	80.25	Positive	0	0	
NC_002041	SBWMV-N	RNA1	JSBWMV	80.5	Negative	0	0	
KT736088	SBWMV-NY	RNA1	JSBWMV	79.25	False positive	0	0	False positive detection likely
NC_002351	SBCMV	RNA1	JSBWMV	82	Negative	1	0	Insertion (3 nt)
AJ132576	SBCMV	RNA1	JSBWMV	82	Negative	1	0	Insertion (3 nt)
NC_002042	SBWMV	RNA2	SBWMV	80	Positive	0	0	
KT736089	SBWMV-NY	RNA2	SBWMV	80	Positive	2	0	
NC_002330	SBCMV	RNA2	SBWMV	81	Negative	3	1	
AJ132577	SBCMV	RNA2	SBWMV	81	Negative	3	1	
NC_038851	JSBWMV	RNA2	SBWMV	81	Negative	4	1	
AJ749657	JSBWMV	RNA2	SBWMV	81	Negative	4	1	
NC_002330	SBCMV	RNA2	SBCMV	80.75	Positive	0	0	
AJ132577	SBCMV	RNA2	SBCMV	80.5	Positive	0	0	
NC_002042	SBWMV-N	RNA2	SBCMV	82	Negative	2	1	
KT736089	SBWMV-NY	RNA2	SBCMV	82	Negative	2	1	
NC_038851	JSBWMV	RNA2	SBCMV	81	Negative	1	2	
AJ749657	JSBWMV	RNA2	SBCMV	74/81	Negative	1	2	Double peak predicted
NC_038851	JSBWMV	RNA2	JSBWMV	79	Positive	0	0	
AJ749657	JSBWMV	RNA2	JSBWMV	79	Positive	0	0	
NC_002042	SBWMV-N	RNA2	JSBWMV	79	False Positive	0	3	Primer binding and amplicon formation unlikely
KT736089	SBWMV-NY	RNA2	JSBWMV	79	False Positive	0	3	Primer binding and amplicon formation unlikely
NC_002330	SBCMV	RNA2	JSBWMV	81	Negative	2	2	Insertion (3 nt)
AJ132577	SBCMV	RNA2	JSBWMV	81	Negative	2	2	Insertion (3 nt)

The specificity of the PCR was assessed by predicting the melting temperature (Tm) of the PCR amplicon, which should be amplified with the respective target and primers by HRM. Based on the rule that samples were considered negative when the difference measured between the amplicon melting temperature and the melting temperature obtained for the standard (see Table 2) exceeded one degree Celsius, the PCR result was extrapolated. In the above table, mismatch 5 LNT FWD is the number of mismatches that occurred between the five 3′ proximal nucleotides upon alignment between virus and forward primer sequences, and mismatch 5 LNT REV is the number of mismatches that occurred between the five 3′ proximal nucleotides upon alignment between virus and reverse primer sequences.

## Data Availability

The raw data supporting the conclusions of this article will be made available by the authors upon request.

## Supplementary Materials

The following supporting information can be downloaded at https://www.mdpi.com/article/10.3390/v16101579/s1, Figure S1: Standard curves for virus quantification; Figure S2: Nucleotide alignment showing the genericity of the qPCR primers for RNA1 of different isolates of the furoviruses; Figure S3: Nucleotide alignment showing the genericity of the qPCR primers for RNA2 of different isolates of the furoviruses; Figure S4: Nucleotide alignment showing the specificity of the RT-qPCR primers for RNA1 of the different furoviruses; Figure S5: Nucleotide alignment showing the specificity of the RT-qPCR primers for RNA2 of the different furoviruses; Table S1: Sequences retrieved from public databases to draw primers to clone and quantify RNA1 and RNA2 from SBWMV, SBCMV and JSBWMV; Table S2: Primers used to clone RNA1 and RNA2 from SBWMV, SBCMV and JSBWMV; Table S3: Primers used in real-time RT-PCR to amplify RNA1 and RNA2 from SBWMV, SBCMV and JSBWMV.
