# Supplementary material for: Detection and Quantification of Soil-Borne Wheat Mosaic Virus, Soil-Borne Cereal Mosaic Virus and Japanese Soil-Borne Wheat Mosaic Virus by ELISA and One-Step SYBR Green Real-Time Quantitative RT-PCR
Source: Viruses. 2024 Oct 8;16(10):1579. doi: 10.3390/v16101579 (PMC11512275; doi:10.3390/v16101579)
Supplement: Supplementary file 1 [file viruses-16-01579-s001.zip › viruses-3195789-supplementary.pdf]

# Supplementary Material

# Detection and Quantification of Soil-Borne Wheat Mosaic Virus, Soil-Borne Cereal Mosaic Virus and Japanese Soil-Borne Wheat Mosaic Virus by ELISA and One-Step SYBR Green Real-Time Quantitative RT-PCR

**Kevin Gauthier [1,2], Claudia Janina Strauch [1], Sabine Bonse [1], Petra Bauer [1], Carolin Heidler [1] and Annette Niehl [1,*]**

[1] Julius Kühn Institute (JKI)-Federal Research Centre for Cultivated Plants, Institute for Epidemiology and Pathogen Diagnostics, Messeweg 11-12, 38104 Brunswick, Germany
[2] Agroscope, Department of Plant Breeding, Route de Duillier 60, 1260 Nyon, Switzerland
* Correspondence: annette.niehl@julius-kuehn.de

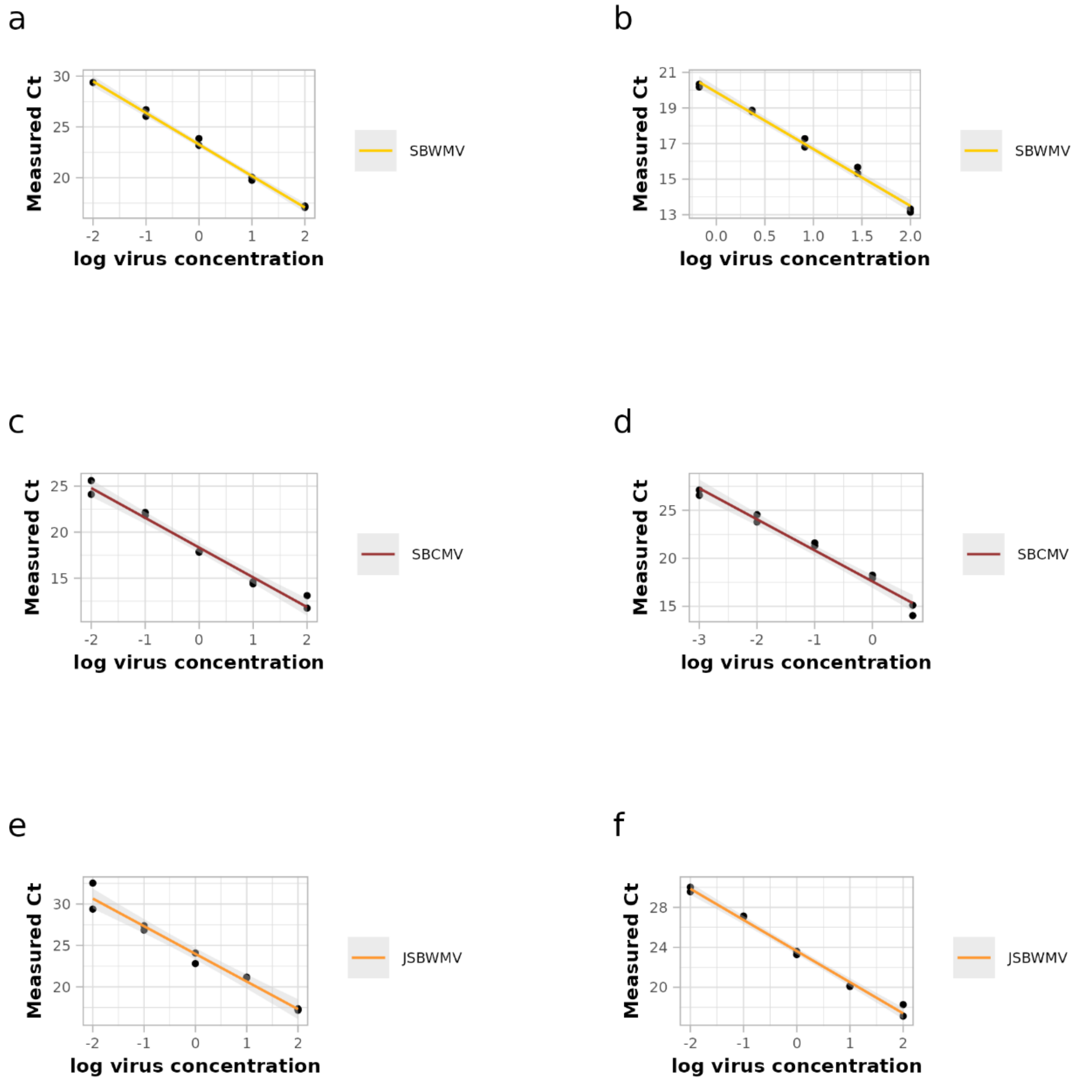


Figure S1. Standard curves for virus quantification. Standard curves for the quantification of SBWMV RNA1 (a) and RNA2 (b), SBCMV RNA1 (c) and RNA2 (d) and JSBWMV RNA1 (e) and RNA2 (f). Measured Ct: Number of amplification cycles required to exceed the detection threshold. Log virus concentration is in ng virus RNA $\mu l^{-1}$ total RNA. Confidence intervals are based on Student's distribution approximation and represented by grey zones.

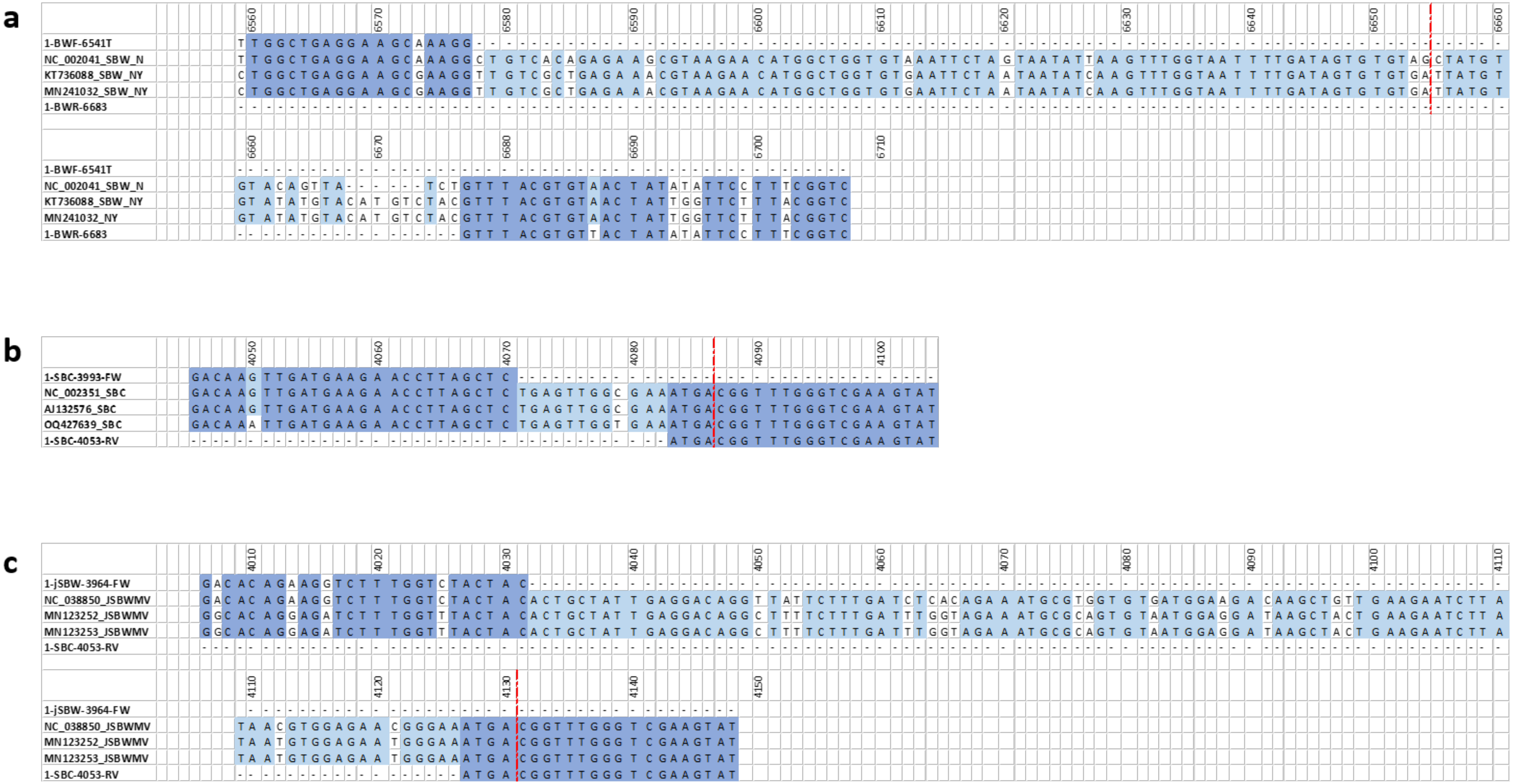

Figure S2. Nucleotide alignment showing the genericity of the qPCR primers for RNA1 of different isolates of the furoviruses. (a), SBWMV; (b), SBCMV; and (c), JSBWMV. Numbers above the alignment indicate the position of the nucleotides in the reference genome of the virus targeted by the primers after alignment. Blue coloration indicates the percentage of identity for each nucleotide between all presented sequences. White: <50%, light blue: between 50 and 62.5%,

medium blue: between 62.5 and 75%, dark blue: >75%. – indicates a missing nucleotide after alignment. The names of SBWMV, SBCMV and JSBWMV primers and isolates shown on the left. ORF limits are presented by red vertical lines.

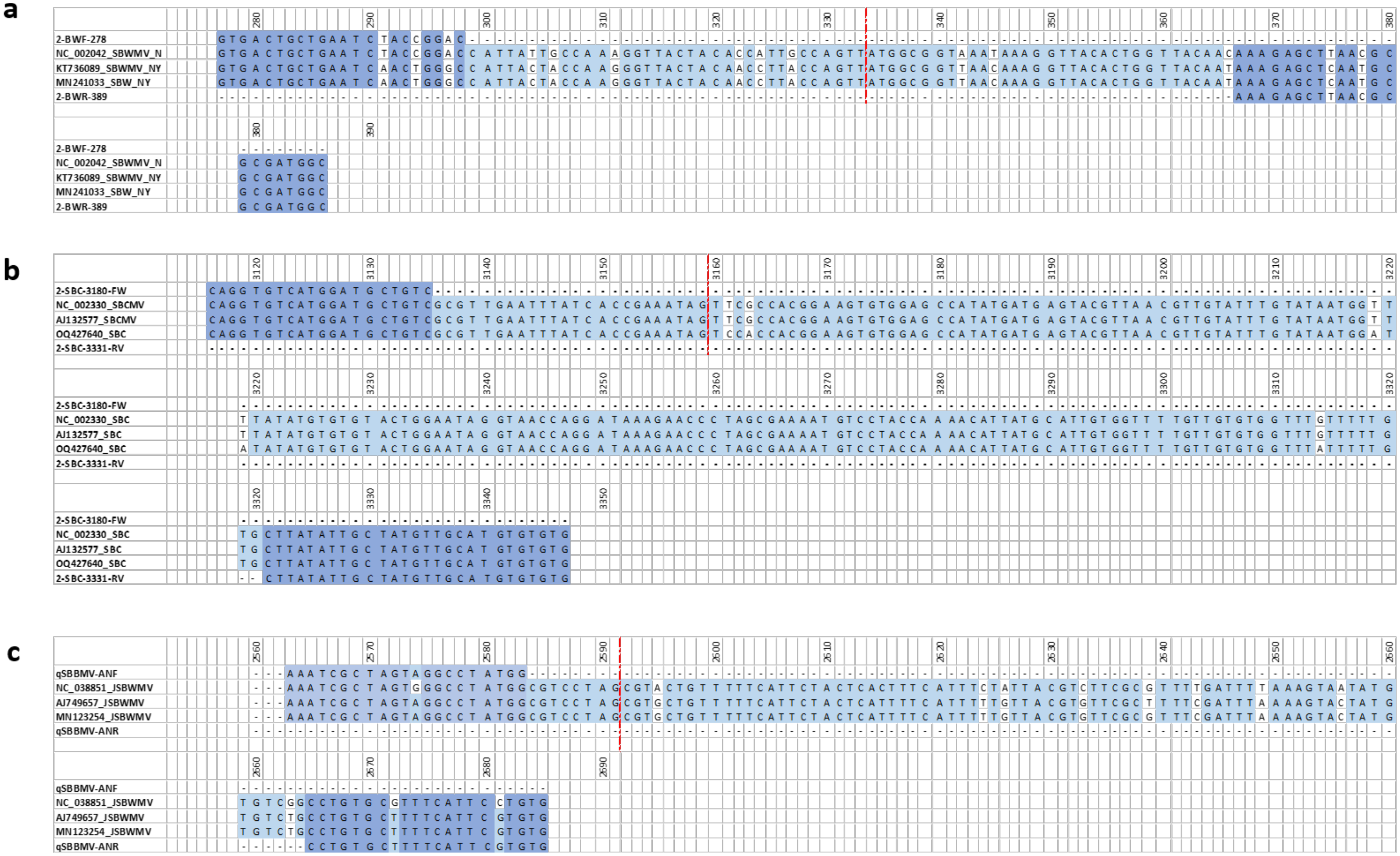
a
2-BWF-278
NC_002042_SBWMV_N
KT736089_SBWMV_NY
MN241033_SBW_NY
2-BWR-389
b
2-SBC-3180-FW
NC_002330_SBCMV
AJ132577_SBCMV
OQ427640_SBC
2-SBC-3331-RV
NC_002330_SBC
AJ132577_SBC
c
qSBBMV-ANF
NC_038851_JSBWMV
AJ749657_JSBWMV
MN123254_JSBWMV
qSBBMV-ANR

Figure S3. Nucleotide alignment showing the genericity of the qPCR primers for RNA2 of different isolates of the furoviruses. (a), SBWMV; (b), SBCMV; and (c), JSBWMV. Numbers above the alignment indicate the position of the nucleotides in the reference genome of the virus targeted by the primers after alignment. Blue coloration indicates the percentage of identity for each nucleotide between all presented sequences. White: <50%, light blue: between 50 and 62.5%, medium blue: between 62.5 and 75%, dark blue: >75%. – indicates a missing nucleotide after alignment. The names of SBWMV, SBCMV and JSBWMV primers and isolates shown on the left. ORF limits are presented by red vertical lines.

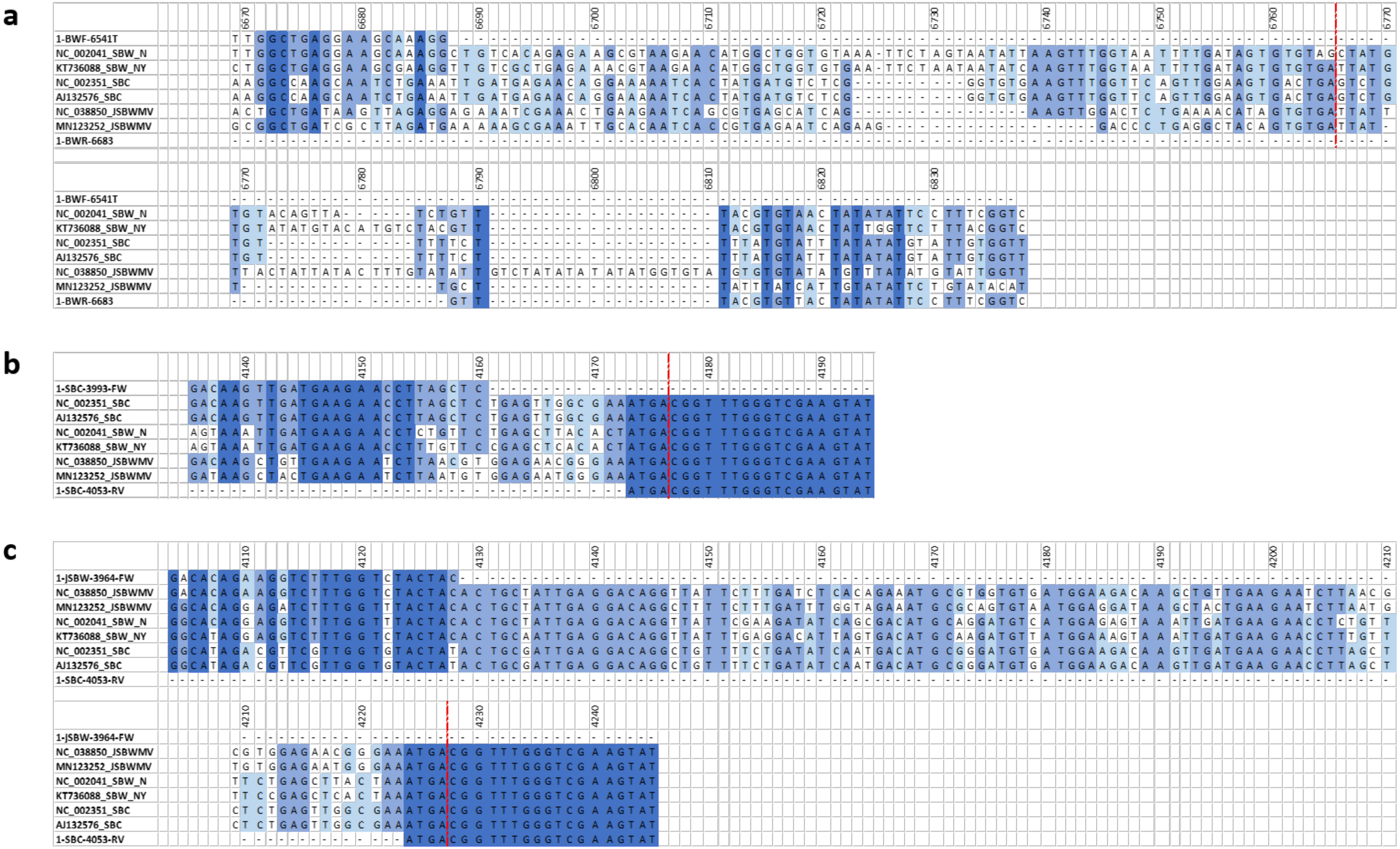
a
6670
6680
6690
6700
6710
6720
6730
6740
6750
6760
6770
1-BWF-6541T
NC_002041_SBW_N
KT736088_SBW_NY
NC_002351_SBC
AJ132576_SBC
NC_038850_JSBWMV
MN123252_JSBWMV
1-BWR-6683
6770
6780
6790
6800
6810
6820
6830
b
4140
4150
4160
4170
4180
4190
1-SBC-3993-FW
NC_002351_SBC
AJ132576_SBC
NC_002041_SBW_N
KT736088_SBW_NY
NC_038850_JSBWMV
MN123252_JSBWMV
1-SBC-4053-RV
c
4110
4120
4130
4140
4150
4160
4170
4180
4190
4200
4210
1-JSBW-3964-FW
NC_038850_JSBWMV
MN123252_JSBWMV
NC_002041_SBW_N
KT736088_SBW_NY
NC_002351_SBC
AJ132576_SBC
1-SBC-4053-RV
4210
4220
4230
4240

Figure S4. Nucleotide alignment showing specificity of the RT-qPCR primers for RNA1 of the different furoviruses. (a), SBWMV; (b), SBCMV; (c), JSBWMV. Numbers above the alignment indicate the position of the nucleotides in the reference genome of the virus targeted by the primers after alignment. Blue coloration indicates the percentage of identity for each nucleotide between all presented sequences. White: <50%, light blue: between 50 and 62.5% medium blue: between 62.5 and 75%, dark blue: >75%. – indicates a missing nucleotide after alignment. The names of SBWMV, SBCMV and JSBWMV primers and isolates are shown on the left. ORF limits are presented by red vertical lines.

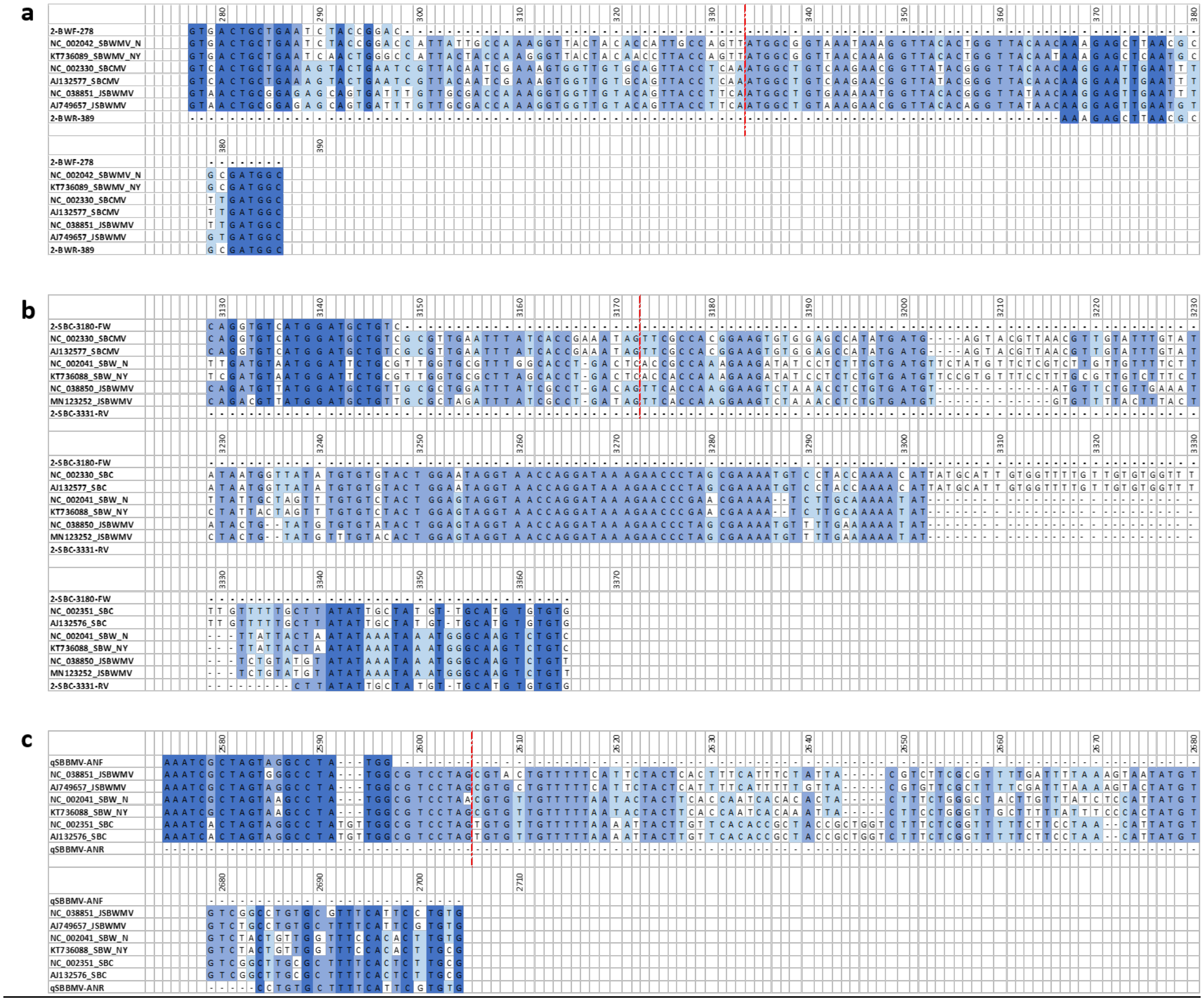
a
2-BWF-278
NC_002042_SBWMV_N
KT736089_SBWMV_NY
NC_002330_SBCMV
AJ132577_SBCMV
NC_038851_JSBWMV
AJ749657_JSBWMV
2-BWR-389
b
2-SBC-3180-FW
NC_002330_SBCMV
AJ132577_SBCMV
NC_002041_SBW_N
KT736088_SBW_NY
NC_038850_JSBWMV
MN123252_JSBWMV
2-SBC-3331-RV
c
qSBBMV-ANF
NC_038851_JSBWMV
AJ749657_JSBWMV
NC_002041_SBW_N
KT736088_SBW_NY
NC_002351_SBC
AJ132576_SBC
qSBBMV-ANR

Figure S5. Nucleotide alignment showing specificity of the RT-qPCR primers for RNA2 of the different furoviruses. (a), SBWMV; (b), SBCMV; (c), JSBWMV. Numbers above the alignment indicate the position of the nucleotides in the reference genome of the virus targeted by the primers after alignment. Blue coloration indicates the percentage of identity for each nucleotide between all presented sequences. White: <50%, light blue: between 50 and 62.5% medium blue: between 62.5 and 75%, dark blue: >75%. – indicates a missing nucleotide after alignment. The names of SBWMV, SBCMV and JSBWMV primers and isolates are shown on the left. ORF limits are presented by red vertical lines.

Table S1. Sequences retrieved from public databases to draw primers to clone and quantify RNA1 and RNA2 from SBWMV, SBCMV and JSBWMV.

| Virus species | RNA | Reference |
|---|---|---|
| SBWMV | RNA1 | NC_002041.1 |
| SBWMV | RNA1 | KT736088.1 |
| SBWMV | RNA1 | L07937.1 |
| SBWMV | RNA1 | AF519798.1 |
| SBWMV | RNA1 | AF361641.1 |
| SBWMV | RNA1 | AY016007.1 |
| SBWMV | RNA1 | MN241032.1 |
| SBWMV | RNA1 | HM133584.1 |
| SBWMV | RNA1 | KU193757.1 |
| SBWMV | RNA1 | KU193756.1 |
| SBWMV | RNA1 | KU193755.1 |
| SBWMV | RNA1 | KU193754.1 |
| SBWMV | RNA1 | KU193753.1 |
| SBWMV | RNA1 | KU193752.1 |
| SBWMV | RNA1 | KU193751.1 |
| SBWMV | RNA1 | KU193750.1 |
| SBWMV | RNA1 | KU193749.1 |
| SBWMV | RNA2 | NC_002042.1 |
| SBWMV | RNA2 | KT736089.1 |
| SBWMV | RNA2 | L07938.1 |
| SBWMV | RNA2 | KX825886.1 |
| SBWMV | RNA2 | EU826170.1 |
| SBWMV | RNA2 | AF361642.1 |
| SBWMV | RNA2 | AY016008.1 |
| SBWMV | RNA2 | JX468082.1 |
| SBWMV | RNA2 | JX468081.1 |
| SBWMV | RNA2 | AF519800.1 |
| SBWMV | RNA2 | AF519799.1 |
| SBWMV | RNA2 | D86320.1 |
| SBWMV | RNA2 | AB002813.1 |
| SBWMV | RNA2 | AB002812.1 |
| SBWMV | RNA2 | X89078.1 |
| SBWMV | RNA2 | X81639.1 |
| SBWMV | RNA2 | MN241033.1 |
| SBWMV | RNA2 | HM133583.1 |
| SBWMV | RNA2 | KU200256.1 |
| SBWMV | RNA2 | KU200255.1 |
| SBWMV | RNA2 | KU200254.1 |
| SBWMV | RNA2 | KU193760.1 |
| SBWMV | RNA2 | KU193759.1 |
| SBWMV | RNA2 | KU193758.1 |
| SBWMV | RNA2 | KX852456.1 |
| SBWMV | RNA2 | KX852455.1 |
| SBWMV | RNA2 | KX852454.1 |
| SBWMV | RNA2 | KX852453.1 |
| SBCMV | RNA1 | NC_002351.1 |
| SBCMV | RNA1 | AJ132576.1 |

| | | |
|---|---|---|
| SBCMV | RNA1 | AF183160.1 |
| SBCMV | RNA1 | AF183162.1 |
| SBCMV | RNA1 | AF183163.1 |
| SBCMV | RNA1 | AF183165.1 |
| SBCMV | RNA1 | AF183168.1 |
| SBCMV | RNA1 | AJ298068.1 |
| SBCMV | RNA2 | NC_002330.1 |
| SBCMV | RNA2 | FN298366.1 |
| SBCMV | RNA2 | FN298365.1 |
| SBCMV | RNA2 | FN298364.1 |
| SBCMV | RNA2 | FN298363.1 |
| SBCMV | RNA2 | FN298362.1 |
| SBCMV | RNA2 | AF183171.1 |
| SBCMV | RNA2 | AF183170.1 |
| SBCMV | RNA2 | AF183167.1 |
| SBCMV | RNA2 | AF183166.1 |
| SBCMV | RNA2 | AF183164.1 |
| SBCMV | RNA2 | AF183161.1 |
| SBCMV | RNA2 | AJ298069.1 |
| SBCMV | RNA2 | AJ298070.1 |
| SBCMV | RNA2 | AJ132577.1 |
| JSBWMV | RNA1 | NC_038850.1 |
| JSBWMV | RNA1 | MN123252.1 |
| JSBWMV | RNA1 | MN123253.1 |
| JSBWMV | RNA2 | NC_838851.1 |
| JSBWMV | RNA2 | AJ749657 |
| JSBWMV | RNA2 | MN123254.1 |

Table S2. Primers used to clone RNA1 and RNA2 from SBWMV, SBCMV and JSBWMV.

| Virus | RNA | Primer name | Size (bases) | Primer direction | Primer sequence |
|---|---|---|---|---|---|
| SBWMV | RNA1 | 1-BWF-6237[§] | 781 | Forward | GTTGGGTTATGATAAAAGTCTGAAGATG |
| SBWMV | RNA1 | 1-BWR-7018[§] | | Reverse | CCTCATCATCGCTAAATGTTGATCT |
| SBCMV | RNA1 | 1-SBC-3658-FW | 1478 | Forward | CATTGTGTTCCCTACCAGCG |
| SBCMV | RNA1 | 1-SBC-5136-RV | | Reverse | ACACGGATCCTCAATGCAGA |
| JSBWMV | RNA1 | 1-jSBW-3964-FW | 141 | Forward | GACACAGAAGGTCTTTGGTCTACTAC |
| JSBWMV | RNA1 | 1-SBC-4053-RV | | Reverse | ATACTTCGACCCAAACCGTCAT |
| SBWMV | RNA2 | 2_BWF_212 | 915 | Forward | CGCTGGATAAGTTTGCTAGACTCA |
| SBWMV | RNA2 | 2_BWR_1127 | | Reverse | TGACTATTACCATCTCCACATCTTCAG |
| SBCMV | RNA2 | SBC2-5-2565[*] | 1049 | Forward | CACTAGTAGGCCTATGTTGGCG |
| SBCMV | RNA2 | SBC2-3-3614[*] | | Reverse | GACCACCAGTCGCTCCTCATCAT |
| JSBWMV | RNA2 | qSBBMV-ANF | 123 | Forward | AAATCGCTAGTAGGCCTATGG |
| JSBWMV | RNA2 | qSBBMV-ANR | | Reverse | CACACGAATGAAAAGCACAGG |

**:* Ziegler, A.; Klingebeil, K.; Papke, V.; Kastirr, U. Quantification of Wheat Spindle Streak Mosaic Virus and Soil Borne Cereal Mosaic Virus in Resistance Testing of Field Samples of Triticale Using Real-Time RT-PCR. *J Plant Dis Prot* **2014**, *121*, 149–155, doi:10.1007/BF03356503.

[§]. Gauthier, K., Pankovic, D., Nikolic, M., Hobert, M., Germeier, C.U., Ordon, F., Perovic, D., Niehl, A. Nutrients and soil structure influence furovirus infection of wheat. Front Plant Sci. 2023 Aug 4;14:1200674. doi: 10.3389/fpls.2023.1200674

Table S3. Primers used in real time RT-PCR to amplify RNA1 and RNA2 from SBWMV, SBCMV and JSBWMV.

| Virus | RNA | Primer name | Size (bases) | Primer direction | Primer sequence |
|---|---|---|---|---|---|
| SBWMV | RNA1 | 1-BWF-6541T[§] | 142 | Forward | TTGGCTGAGGAAGCAAAGG |
| SBWMV | RNA1 | 1-BWR-6683[§] | | Reverse | GACCGAAAGGAATATATAGTAACACGTAAAC |
| SBCMV | RNA1 | 1-SBC-3993-FW | 60 | Forward | GACAAGTTGATGAAGAACCTTAGCTC |
| SBCMV | RNA1 | 1-SBC-4053-RV | | Reverse | ATACTTCGACCCAAACCGTCAT |
| JSBWMV | RNA1 | 1-jSBW-3964-FW | 141 | Forward | GACACAGAAGGTCTTTGGTCTACTAC |
| JSBWMV | RNA1 | 1-SBC-4053-RV | | Reverse | ATACTTCGACCCAAACCGTCAT |
| SBWMV | RNA2 | 2-BWF-278 | 111 | Forward | GTGACTGCTGAATCTACCGGAC |
| SBWMV | RNA2 | 2-BWR-389 | | Reverse | GCCATCGCGTTAAGCTCTTT |
| SBCMV | RNA2 | 2-SBC-3180-FW | 151 | Forward | CAGGTGTCATGGATGCTGTC |
| SBCMV | RNA2 | 2-SBC-3331-RV | | Reverse | CACACACATGCAACATAGCAATATAAG |
| JSBWMV | RNA2 | qSBBMV-ANF | 123 | Forward | AAATCGCTAGTAGGCCTATGG |
| JSBWMV | RNA2 | qSBBMV-ANR | | Reverse | CACACGAATGAAAAGCACAGG |

[§]. Gauthier, K., Pankovic, D., Nikolic, M., Hobert, M., Germeier, C.U., Ordon, F., Perovic, D., Niehl, A. Nutrients and soil structure influence furovirus infection of wheat. Front Plant Sci. 2023 Aug 4;14:1200674. doi: 10.3389/fpls.2023.1200674
